# Supplementary material for: Genome-Wide Analysis of WRKY Transcription Factors Involved in Abiotic Stress and ABA Response in Caragana korshinskii
Source: Int J Mol Sci. 2023 May 30;24(11):9519. doi: 10.3390/ijms24119519 (PMC10253768; doi:10.3390/ijms24119519)
Supplement: Supplementary file 1 [file ijms-24-09519-s001.zip › S4-Gene name of Ortholog in C. intermedia.pdf]

Table S4. Gene name of Ortholog in C. intermedia.

**New name of gene**	**Published *CkWRKYs***	**Ortholog in *C. intermedia* [1]**
CkWRKY1	-	-
CkWRKY2	-	*CiWRKY12*[2]
CkWRKY3	-	*CiWRKY30*[3]
CkWRKY4	-	-
CkWRKY5	-	-
CkWRKY6	-	*CiWRKY36*
CkWRKY7	-	*CiWRKY32-1*
CkWRKY8	-	-
CkWRKY9	-	-
CkWRKY10	-	*CiWRKY48*[4]
CkWRKY11	-	-
CkWRKY12	-	*CiWRKY2*
CkWRKY13	-	-
CkWRKY14	-	-
CkWRKY15	-	-
CkWRKY16	-	-
CkWRKY17	-	-
CkWRKY18	-	-
CkWRKY19	-	*CiWRKY70-1*
CkWRKY20	-	-
CkWRKY21	-	-
CkWRKY22	-	*CiWRKY40-4/ 45*[5]
CkWRKY23	-	-
CkWRKY24	-	*CiWRKY23*
CkWRKY25	-	-
CkWRKY26	-	-
CkWRKY27	-	*CiWRKY17*[6]
CkWRKY28	-	-
CkWRKY29	-	*CiWRKY40-3*
CkWRKY30	-	*CiWRKY50*
CkWRKY31	-	-
CkWRKY32	-	-
CkWRKY33	-	-
CkWRKY34		*CiWRKY41-1*
CkWRKY35	CkWRKY1[7]	*CiWRKY40-1*
CkWRKY36	-	-
CkWRKY37	-	*CiWRKY51*
CkWRKY38	-	*CiWRKY28-1*[8]
CkWRKY39	-	-
CkWRKY40	-	-
CkWRKY41	-	*CiWRKY6-1*
CkWRKY42	-	-
CkWRKY43	-	
CkWRKY44	-	-
CkWRKY45	-	-
CkWRKY46	-	-
CkWRKY47	-	-
CkWRKY48	-	-

CkWRKY49	-	-
CkWRKY50	*CkWRKY33*[9]	*CiWRKY33-1*
CkWRKY51	-	-
CkWRKY52	-	*CiWRKY75-1*[10]/ *6*[5]
CkWRKY53	-	-
CkWRKY54	-	-
CkWRKY55	-	-
CkWRKY56	-	-
CkWRKY57	-	-
CkWRKY58	-	-
CkWRKY59	-	-
CkWRKY60	-	*CiWRKY69-1*
CkWRKY61	-	-
CkWRKY62	-	-
CkWRKY63	-	-
CkWRKY64	-	-
CkWRKY65	-	-
CkWRKY66	-	-
CkWRKY67	-	*CiWRKY6-2*
CkWRKY68	-	-
CkWRKY69	-	-
CkWRKY70	-	-
CkWRKY71	-	*CiWRKY28-2*
CkWRKY72	-	*CiWRKY26*[8]/ *2*[11]
CkWRKY73	-	-
CkWRKY74	-	-
CkWRKY75	-	-
CkWRKY76	-	*CiWRKY57*
CkWRKY77	-	*CiWRKY3-1*
CkWRKY78	-	*CiWRKY15*[12]
CkWRKY79	-	-
CkWRKY80	-	-
CkWRKY81	-	-
CkWRKY82	-	-
CkWRKY83	-	-
CkWRKY84	-	-
CkWRKY85	-	*CiWRKY3-2*
CkWRKY86	-	-

Reference

1. Wan, Y., et al., Identification of the WRKY gene family and functional analysis of two genes in *Caragana intermedia*. BMC Plant Biol, 2018. 18(1): p. 31.
2. Wan, D., et al., Cloning and Bioinformatics Analysis of *Caragana intermedia WRKY12* gene. Molecular Plant Breeding, 2020. 18(1): p. 9.
3. Liu, J., et al., Cloning and Bioinformatics Analysis of *Caragana interm*edia *WRKY30*. Molecular Plant Breeding, 2019. 17(14): p. 10.
4. Liu, J., et al., Cloning and prokaryotic expression of *WRKY48* from *Caragana intermedia*. Open Life Sci, 2022. 17(1): p. 131-138.
5. Mao, M., Functional analysis of Two *WRKY* Genes in *Caragana intermedia*. 2017, Inner Mongolia Agricultural University.
6. Bai, M., et al., Cloning and Bioinformatics Analysis of *CiWRKY17* Gene. Genomics and Applied Biology, 2020. 39(8): p. 9.

7. Yang, Q., Construction of a Suppression Subtractive Hybridization Library of *Caragana korshinskii* Under Drought Stress and Cloning of *CkWRKY1* Gene. SCIENTIA SILVAE SINICAE, 2013. 49(7): p. 62-68.
8. Wan, Y., Identification of the WRKY Gene Family and Functional Analysis of Two CiWRKYs in *Caragana intermedia*. 2018, Inner Mongolia Agricultural University.
9. Li, Z., et al., Enhanced tolerance to drought stress resulting from *Caragana korshinskii* CkWRKY33 in transgenic *Arabidopsis thaliana*. BMC Genom Data, 2021. 22(1): p. 11.
10. Wan, Y., et al., *Caragana intermedia* WRKY75 Altered *Arabidopsis thaliana* Tolerance to Salt Stress and ABA. Acta Botanica Boreali-Occidentalia Sinica, 2018. 38(1): p. 9.
11. Mao, M., et al., Cloning and Expression Analysis of *CiWRKY2* Gene in *Caragana intermedia*. Molecular Plant Breeding, 2017. 15(4): p. 9.
12. Zhang, W., et al., Cloning and Bioinformatics Analysis of *Caragana intermedia WRKY15*. Chinese Journal of Grassland, 2019(4): p. 9.
